# Operation for Ancient Dislocation of Elbow and Bridge of Callus between Radius and Ulna

**Published:** 1894-06

**Authors:** John B. Roberts

**Affiliations:** Philadelphia, Pa.


### OPERATION FOR ANCIENT DISLOCATION OF ELBOW AND BRIDGE OF CALLUS BETWEEN RADIUS AND ULNA.

BY JOHN B. ROBERTS, M.D., PHILADELPHIA, PA.

The patient had an old dislocation of the elbow, which occurred two years ago, and was the result of a fall from a carriage, which was overturned. The injury was evidently a backward dislocation of both bones of the forearm, with compound fracture of the radius at the junction of the upper and middle third. The injury occurred two years before the patient, who is exhibited, came under my care at the Woman's Hospital. She at that time had the right arm rigidly extended with no motion at the elbow; suffered with pain and numbness in the fingers at the ulna side of the hand, due to pressure on the ulna nerve, and had a suppurating sinus at the point of depression, shown in this cast made of the arm before operation.

It seemed to me that it was proper to attempt to treat the old dislocation, although of two years' standing, by making a resection in order to get the arm in a flexed position. I cut down upon the olecranon, and found that the head of the radius and the olecranon were soldered by callus, to the humerus, in the abnormal position. The ulna nerve was displaced and in a condition of tension. I was compelled to chisel loose the radius and ulna and cut off the triceps tendon in order to put the bones at the elbow in the position of flexion, which would be so much more useful and convenient, even if the elbow were immovable. In order to unite the triceps tendon to the ulna after flexing the joint, I was obliged to lengthen it by cutting a V-shaped flap out of the tendon. This I did with its apex upward, and then turned the flap over with its point downward, and sutured it to the stump which had been left attached to the olecranon. I used silk sutures. I lessened the tension on the ulna nerve by replacing it; in the course of a few days the numbness of the fingers disappeared.

In order to cover in the gap in the skin, I made a large plastic operation by dissecting a flap from the forearm. Her arm was now flexed at a right angle, and in the course of, perhaps, two months, I got a considerable degree of motion. The radius and ulna were still united at the seat of the sinus by a bridge of callus, which prevented pronation and supination. I therefore determined to cut down and chisel out the bridge of bone and see if I could establish pronation and supination. Upon cutting down I found a sequestrum due to necrosis of the radius at the point of fracture. I chiselled away a considerable amount of bone and took out the dead portion of the radius. Knowing that the head of the radius would not be likely to rotate, as it had no cartilage upon it, I determined to make an artificial joint in order to get some pronation and supination in addition to the amount of flexion already obtained at the elbow. I excised three-quarters of an inch of the radius, and then encouraged the girl, after I had removed the portion of bone and the wound had partly healed, to make motions of rotation of the hand.

She obtained a certain amount of *passive* supination and pronation, and the fingers became more flexible. During our endeavors at making passive extension and flexion of the elbow, fracture of the ulna took place, some weeks later, in the upper third of the shaft. This necessitated putting her arm in a splint, and prevented our continuing with the massage and other motions to get motion at the elbow and wrist. As the elbow would probably become stiff, I, in order to get the hand more toward the face, allowed the fragments to unite with a little angularity. You see now a bend or angular deformity at the seat of fracture, which enables her to bring the hand nearer the mouth than otherwise would have been possible. The wound has not entirely healed. She lost nearly all the flexion and extension she had from the first operation during immobilization of the joint for repair of the fracture of the ulna. She has a little motion at the elbow, not enough to be useful; there is practically no supination or pronation of the hand, but the numbness of the fingers is gone, and she can bend her fingers, which were stiff, pretty well. She has, and had, good motion at the wrist. We have not gained a great deal, therefore, except the right-angle position of the elbow, motion of the fingers, and freedom from pain and numbness. If fracture had not taken place I think we would have obtained considerable motion at the elbow and have been able to maintain the false joint at the point of radial resection so as to give her some rotation of the hand.

The case is interesting to me because of the lengthening of the tendon of the triceps, the attempt to establish a point of motion in the shaft of the radius, when the head is adherent by ankylosis to the humerus, and the ease with which bridges of callus uniting the radius and ulna can be removed.

